# Heterogeneous Evolution of HIV-1 CRF01_AE in Men Who Have Sex with Men (MSM) and Other Populations in China

**DOI:** 10.1371/journal.pone.0143699

**Published:** 2015-12-01

**Authors:** Xiaorong Peng, Haibo Wu, Xiuming Peng, Changzhong Jin, Nanping Wu

**Affiliations:** State Key Laboratory for Diagnosis and Treatment of Infectious Diseases, Collaborative Innovation Center for Diagnosis and Treatment of Infectious Diseases, The First Affiliated Hospital, School of Medicine, Zhejiang University, 310003 Hangzhou, China; Fudan University, CHINA

## Abstract

**Introduction:**

The HIV epidemic in men who have sex with men (MSM) continues to grow in most countries. However, the phylodynamic and virological differences among HIV-1 strains circulating in MSM and other populations are not well characterized.

**Methods:**

Nearly full-length genomes (NFLGs) of the HIV-1 CRF01_AE were obtained from the Los Alamos HIV database. Phylogenetic analyses were conducted using the NFLG, *gag*, *pol and env* genes, using the maximum likelihood method. Selection pressure analyses at the codon level were performed for each gene in the phylogenetic clusters using PAML.

**Results:**

Sequences isolated from MSM in China clustered in Clusters 1 (92.5%) and 2 (85.71%). The major risk factor for Cluster 3 was heterosexual transmission (62.16%). The ratio of non-synonymous to synonymous substitutions in the *env* gene (0.7–0.75) was higher than the *gag* (0.26–0.34) or *pol* (0.21–0.26) genes. In *env* gene, Cluster 1 (4.56×10^-3^subs/site/year) and 2 (6.01×10^-3^subs/site/year) had higher evolutionary rates than Cluster 3 (1.14×10^-3^subs/site/year). Positive selection affected 4.2–6.58% of the amino acid sites in the *env* gene. Two sites (HXB2:136 and 316) evolved similarly in Clusters 1 and 2, but not Cluster 3.

**Conclusion:**

The HIV-1 CRF01_AE in MSM is evolving differently than in other populations.

## Introduction

The HIV epidemic in men who have sex with men (MSM) continues to grow in most countries [1]. More than half of new HIV infections occur among MSM in both the United States of America and the United Kingdom [2, 3]. In China, the proportion of MSM among those newly diagnosed with HIV increased to 29.4% in 2011 [4]. The drivers of the HIV epidemic in MSM are complex; they include increased high-risk behaviors, high risk of transmission through receptive anal intercourse, and a high prevalence within the network of possible sexual contacts [5]. There is an unmet need for studies focusing on the phylodynamics and virology of HIV transmission and acquisition risks for MSM and transmission dynamics within the MSM networks.

The molecular epidemiology of HIV infections in MSM in China was first studied in a small cohort (n = 45) in 2005–2006. In this cohort, the predominant subtype was the US-European origin subtype B virus (71.1%), followed by the CRF01_AE (24.4%), and CRF07_BC (4.4%) subtypes [6]. Four serial cross-sectional surveys in MSM, from 2005 to 2009 suggested that Non-B subtypes increased rapidly in recent years, in particular, CRF01_AE increased from 3.7% in 2005 to 50% in 2009 [7]. A nationwide molecular epidemiological survey in MSM showed that CRF01_AE accounted for 62.1% of infections in China as a whole between 2009 and 2011 [5]. In summary, CRF01_AE has become the predominant strain of the virus in MSM in China.

Recent studies have confirmed that CRF01_AE was introduced from Southeast Asia in the 1990s and has expanded rapidly in China [8]. The CRF01_AE epidemic in China is comprised of multiple genetically distinct clusters that have different risk factors and are epidemic in different geographic regions. However, the evolutionary history of the clusters has not been well characterized.

Here, we have conducted a large-scale phylogenetic analysis of nearly full-length genomes (NFLG) of CRF01_AE strains to infer their evolutionary relationship. The substitution rates of the clusters were estimated using the Bayesian Markov Chain Monte Carlo (MCMC) method. We have also estimated the non-synonymous to synonymous substitution rate ratios (dN/dS ratio), and identified the positive selection sites for each cluster. These studies provide novel insights into the evolution of CRF01_AE in MSM, and will likely contribute to improving HIV-1 surveillance and vaccine development.

## Materials and Methods

### Sequence Data

All of the available NFLG sequences for CRF01_AE were obtained from the Los Alamos HIV database (http://www.hiv.lanl.gov/), on April 18, 2015. Identical sequences in the dataset are represented by the oldest sequence in the group. The dataset included 685 sequences. An initial alignment of the sequences was performed using Gene Cutter from the Los Alamos HIV sequence database (http://www.hiv.lanl.gov/content/sequence/GENE_CUTTER/cutter.html). The accession numbers for the sequences used in this study are summarized in S1 File.

### Phylogenetic Tree Analysis

Phylogenetic analysis was performed for the NFLGs, *gag*, *pol* and *env* genes using the maximum likelihood (ML) method in RAxML [9]. Two hundred bootstrap replicates were performed using the GTR-GAMMA, the GTR model of nucleotide substitution with the Gamma model of rate heterogeneity. The tree was color-coded using FigTree (ver.1.4.2) (http://www.tree.bio.ed.ac.uk/software/figtree/).

### Evolutionary Rate

The substitution rates of the different clusters were estimated using the BEAST software and implementing an MCMC method [10]. The GTR+I+Г4 nucleotide substitution model and coalescent Bayesian skyline model were incorporated in the MCMC method [11]. A relaxed molecular clock model with uncorrelated lognormal distribution was used to infer the time-scaled maximum clade credibility phylogenies [12]. Multiple independent MCMC runs were performed and assessed for consistency. The MCMC analyses were combined to give a total chain length of 0.5-4x10^7^ steps with sampling every 5,000 steps. The first 10% of the states of each chain were discarded as burn-in. Ten thousand trees were then sampled to estimate the evolutionary rate using LogCombiner v1.8.0. Convergence of relevant parameters was assessed by effective sample sizes over 200 in Tracer v1.5 (http://tree.bio.ed.ac.uk/software/tracer/).

### Selection Pressure Analysis

To examine the selection pressure placed on each cluster, we estimated the ratio of non-synonymous (dN) to synonymous (dS) substitutions for each cluster, using the HyPhy package [13]. Selection pressure analyses at the codon level for each gene in the different clusters were conducted using the CODEML program in the PAML 4.4 software package to apply site-specific models for detecting positive selection [14]. Two selective models that allow for positive selection (2a and 8; ω>1) were compared with two null models (1a and 7, respectively) that do not allow for positive selection. The likelihood ratio test was used to determine whether there were significant differences between the null model and the alternative model by calculating twice the log-likelihood difference following a χ^2^ distribution, with the number of degrees of freedom [15].

## Results

### Phylogenetic analysis

**Fig 1:**
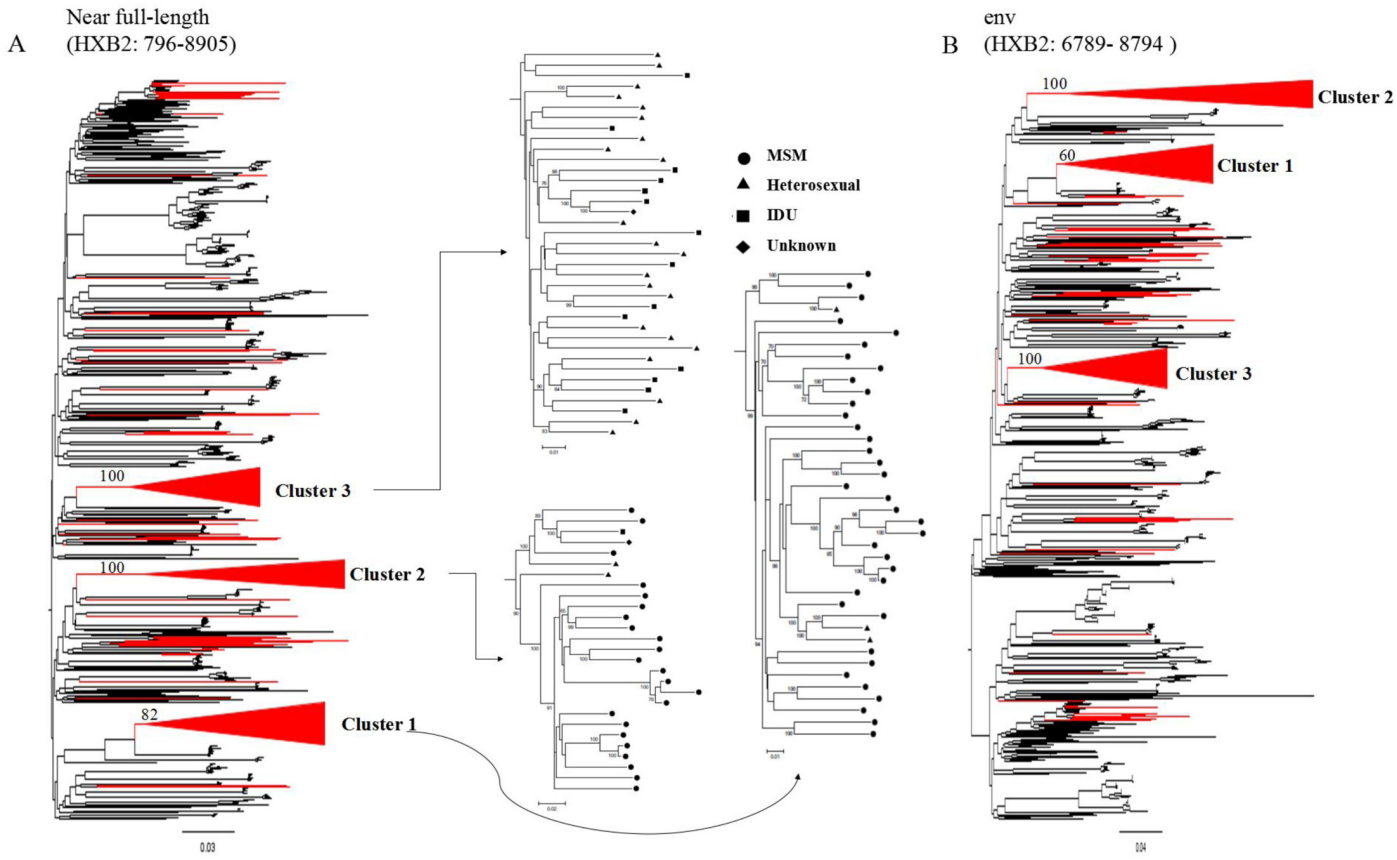
Three distinct phylogenetic clusters of HIV-1 CRF01_AE were identified in China. (A) Phylogenetic tree for NFLG (HXB2: 796–8905 nt). Individual sequences are indicated by symbols corresponding to their known risk groups: MSM (circle); heterosexual (triangle); intravenous drug use (IDU; square); and unknown high-risk behavior (diamond). (B) Phylogenetic tree of the *env* gene (HXB2: 6789–8794 nt). The sequences isolated in China are highlighted in red.

Three phylogenetic trees were constructed from the CRF01_AE NFLGs (HXB2 nucleotide sequence numbering 796 to 8,905 nucleotides [nt]) using the ML approach with bootstrapping analyses to assess clade robustness: (1) *env* fragments (HXB2 6,789 to 8794 nt); (2) *gag* fragments (HXB2 796 to 2,216 nt); and (3) *pol* fragments (HXB2 2,085 to 5,094 nt). The results for the NFLGs and *env* fragments are shown in Fig 1. The results for *pol* and *gag* fragments are shown in S1 Fig. Three clusters (numbered 1, 2, and 3) were observed in CRF01_AE sequences isolated from Chinese patients. MSM was the predominant risk factor for patients in Cluster 1 (92.5%) and 2 (85.71%). In contrast, heterosexuality was the major risk factor for patients in cluster 3 (62.16%).

Cluster 1 (bootstrap support in NFLG [82%] and *env* [60%]) contained 40 sequences that were isolated between 2007 and 2012; notably, 47.5% of the sequences were isolated in 2009. Twenty-six sequences (65%) were isolated in LiaoNing (Table 1).

**Table 1:** Characteristics of HIV-1 CRF01_AE strains in different clusters.

	Number	Risk factors^a^	Province	Sampling Year	
Cluster 1	40	**MSM** (92.5%),	**LiaoNing**(65%)	2007–2012	**2009** (47.5%),
		Hetero (7.5%)			2010 (35%)
Cluster 2	27	**MSM** (85.71%)	**BeiJing** (40.7%),	2007–2011	**2010** (59.2%),
			LiaoNing (25.9%)		2009 (18.5%),
					2007 (14.8%)
Cluster 3	37	**Hetero** (62.16%),	**GuangXi** (48.6%),	2005–2007	**2007** (70.3%),
		IDU (37.83%)	GuangDong (18.9%)		2005 (24.3%),
					2006 (5.4%)

^a^ Risk factors: MSM, men who have sex with men; Hetero, heterosexual; IDU, Intravenous Drug User

Bold values are the most common characteristics of each lineage.

Cluster 2 (100% bootstrap support in NELG and *env*) contained 27 sequences isolated between 2007 and 2011. Over half (59.2%) of the sequences were isolated in 2010. Sixteen sequences (40.7%) were isolated in Beijing (Table 1).

Cluster 3 (100% bootstrap support in NELG and *env*) contained 37 sequences isolated between 2005 and 2007. The majority (70.3%) of the sequences were isolated in 2007. Eighteen sequences (48.6%) were isolated in GuangXi (Table 1).

### Evolutionary analysis

To evaluate the evolutionary changes that characterized the different clusters, we calculated the dN/dS ratios and the evolutionary rates of each cluster (Table 2). The dN/dS ratio represents the magnitude of the selective pressure. A higher selective pressure indicates that the gene (or site) is under stronger positive selective pressure for amino acid substitution [16].

**Table 2:** Substitution rates and selective pressure for each HIV-1 gene by phylogenetic cluster.

Fragments	Clusters	Substitution rates (×10^-3^subs/site/year)	95% HPD Interval	dN/dS	95%CI
*gag*	Cluster 1	2.19	0.93–3.56	0.34	0.31–0.38
	Cluster 2	1.92	0.20–3.55	0.26	0.22–0.3
	Cluster 3	2.37	0.29–4.19	0.32	0.28–0.36
*pol*	Cluster 1	1.363	0.66–2.13	0.23	0.21–0.26
	Cluster 2	1.113	0.23–1.96	0.21	0.19–0.24
	Cluster 3	1.772	0.66–2.82	0.26	0.23–0.29
*env*	Cluster 1	**4.56**	**2.23–6.77**	0.72	0.67–0.77
	Cluster 2	**6.011**	**4.06–7.85**	0.75	0.7–0.81
	Cluster 3	1.14	2.8e-3-3.0	0.7	0.65–0.75

The dN/dS ratio for the *env* gene (0.7–0.75) was higher than the *gag* (0.26–0.34) or *pol* (0.21–0.26) genes, indicating greater selective pressure was exerted on *env*. Within the *env* genes, Cluster 1 (4.56×10^-3^subs/site/year) and Cluster 2 (6.01×10^-3^subs/site/year) had higher evolutionary rates than Cluster 3 (1.14×10^-3^subs/site/year; Table 2).

### Site-by-site Analyses

Positive selection usually affects only a few residues in a protein, therefore we used the site-specific model in the PAML package to identify the positively selected sites (PSS) [17]. Two selection models (M2a and M8) fit the data significantly better than the null models that did not incorporate selection (M1a and M7). The models indicated that 4.2%-6.58% of the amino acid sites in the *env* gene appear to be under positive selection (dN/dS ratio: 3.78–5.81) (Table 3).

**Table 3:** Positive selection characteristics for the *env* gene in each cluster.

Date set	Model^a^	2Δℓ^b^	p^c^	dN/dS^d^	Adaptively evolving amino acids^e^
Cluster 1	M1a vs M2a	491.58	<0.001	5.58	27 (4.56%)
	M7 vs M8	527.50	<0.001	5.22	37 (4.73%)
Cluster 2	M1a vs M2a	286.45	<0.001	4.37	25 (4.2%)
	M7 vs M8	310.01	<0.001	3.78	39 (6.58%)
Cluster 3	M1a vs M2a	429.80	<0.001	5.81	27 (4.56%)
	M7 vs M8	223.49	<0.001	5.50	28 (4.7%)

^a^ Model code in PAML (see Methods).

^b^ the likelihood ratio test statistics (2 delta lambda statistics).

^c^ The P values indicate the level of significance with a χ^2^ distribution and degrees of freedom = 2.

^d^ Associated average dN/dS (ω) of positions under positive selection.

^e^ Codons with a high posterior probability (PP > 0.90) that supports the likelihood of a site having a dN/dS > 1.

**Fig 2:**
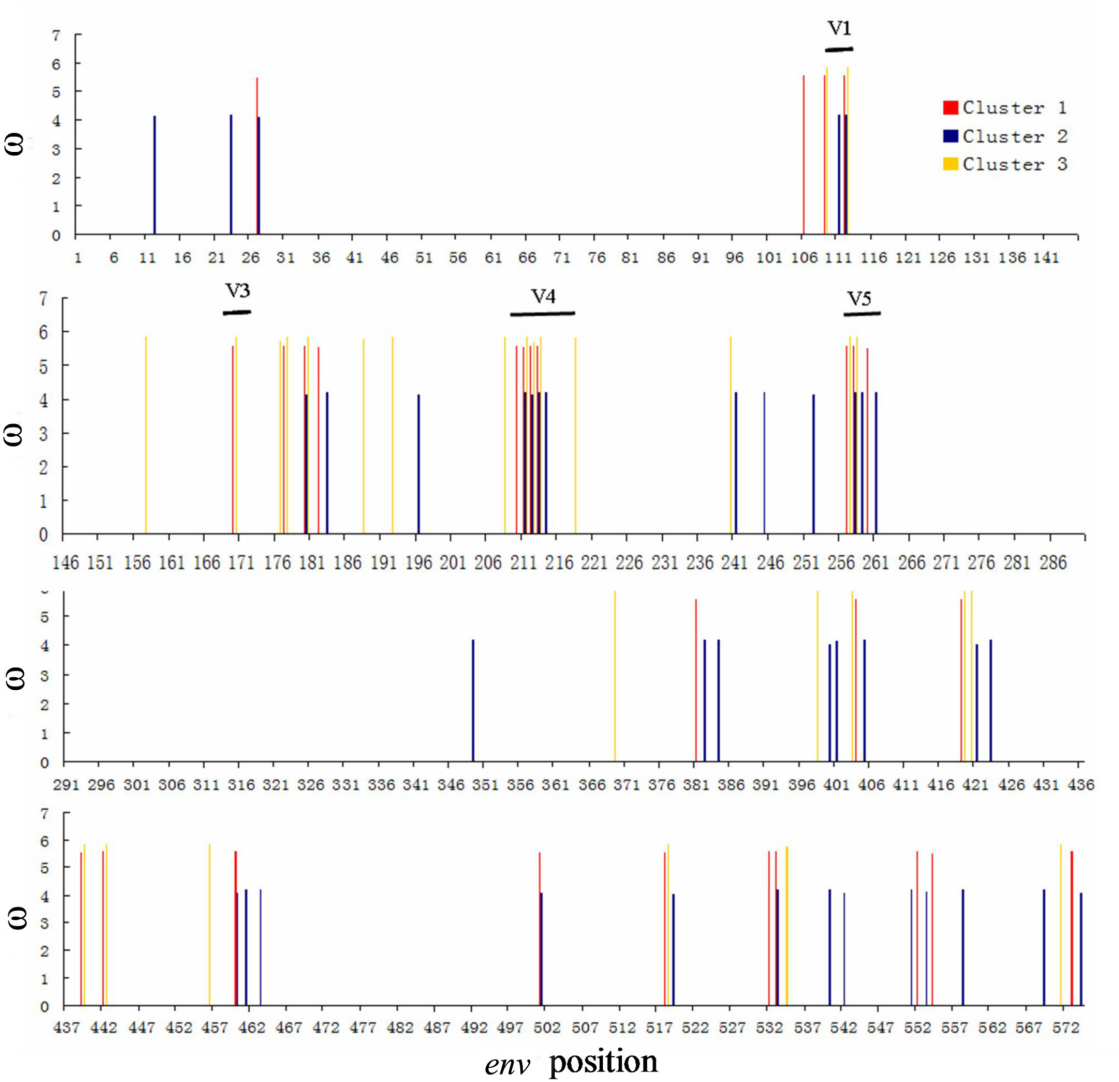
Positively selected sites in the *env* gene from different clusters. The weighted mean dN/dS (ω) value for each site was calculated by multiplying ω by the posterior probability for each cluster under M8 and summing the results. The weighted mean ω ratios of these sites were greater than 1 and the Bayes Empirical Bayes (BEB) probabilities of these sites were higher than 95%. The positions of the five variable regions V1 to V5 are indicated.

We then compared the similarities and differences in the PSS in the three clusters. Four PSS found in clusters 1 and 2 were not under positive selection in cluster 3. Of these, three (HXB2: 721, 775 and 816) were located in gp41, which suggested that the CRF01_AE gp41 protein is evolving and adapting in MSM. Seven PSS were identified in the three clusters that were evolving differently. Of these, two (HXB2:136 and 316) were evolving similarly in Clusters 1 and 2 and were significantly different from Cluster 3 (Fig 2). Threonine (T) was frequently used in site 136 (HXB2) in Clusters 1 (42.5%) and 2 (92.8%), while praline (P) was frequently used in Cluster 3 (52.9%). Lysine (K) was frequently used in site 316 (HXB2) in Clusters 1 (50%) and 2 (96.4%), while asparagine (N) was frequently used in Cluster 3 (47%, Table 4).

**Table 4:** Summary of the amino acids of present at positively selected sites in *env* in each cluster.

	gp120										gp41		
	V1		V3			V4				v5			
HXB2	**136**	139	**316**	340	343	402	403	404	459	464	640	674	677
	**109**	112	**170**	177	180	211	212	213	257	258	419	439	442
Cluster 1	T(42.5%)	D(55%)	K(50%)	E(67.5%)	R(72.5%)	T(80%)	M(42.5%)	E(37.5%)	N(35%)	N(45%)	N(77.5%)	D(80%)	N(72.5%)
Cluster 2	T(92.8%)	G(50%)	K(96.4%)	E(75%)	N(50%)	G(35.7%)	T(82.1%)	M(64.3%)	N(28.6%)	N(60.7%)	N(71.4%)	D(85.7%)	N(78.5%)
Cluster 3	P(52.9%)	K(38.2%)	N(47%)	E(50%)	K(44.1%)	E(41.1%)	T(70.6%)	I(32.3%)	N(44.1%)	N(67.6%)	N(55.9%)	D(70.6%)	N(58.8%)

Bold values indicate positions that are similar in cluster 1 and 2, but markedly different from cluster 3.

## Discussion

In this study, we carried out a large-scale sequence analysis of HIV-1 CRF01_AE. The CRF01_AE sequences isolated from MSM in China formed two clusters similar to previous studies [5]. These findings suggest that MSM have their own group, and that the HIV-1 subtypes circulating in MSM have unique evolutionary characteristics. We also observed distinct differences in the geographical distribution of the clusters: Cluster 1 was found more frequently in LiaoNing, while Cluster 2 was more concentrated in Beijing. As only a small fraction of sequences is included in the database, our study’s dataset is just a small sample of the viruses that circulate worldwide. This is a potential weakness of our study. Sample bias could be one of the reasons causing the distinct geographical differences among clusters.

The selective pressure is stronger on *env* than *gag* or *pol*, and more sites under positive selection were identified in *env*. The *env* gene is associated with viral transmission and host cell tropism; it is also the primary target of the host immune response. Thus, many studies have evaluated its contribution to viral replication and HIV-1 pathogenesis [18]. The selective pressure exerted during transmission enhances *env* entry efficiency and HIV-1 viral fitness, which might help explain the growing epidemic in MSM.

The envelope protein initially forms as a precursor (gp160) that is leaved by a cellular protease to produce the surface subunit gp120 and the transmembrane subunit gp41. The gp120 protein is comprised of five variable (V1 to V5) and five conserved constant (C1 to C5) domains [19]. In our study, many of the PSS were found in the variable domains. However, there were marked differences between clusters. Some PSS were only observed in Clusters 1 and 2 (HBX2: 32, 721, 775, and 816). Sites 136 and 316 (HBX2) evolved differently and are located in the V1 loop and V3 loop respectively. The two sites circulating in MSM preferentially use threonine (T) and Lysine (K).

V1/V2 loop is generally exposed on the envelope and is one of the first targets of the early immune response [18]. Previous studies suggested that more compact or shorter V1/V2s reduce number of N-linked glycosylation sites and increase the number of quasispecies replicating in the plasma of donors at the time of transmission [20]. V1 and V2 also conceal the CD4 binding site; thus, deletion of the V1 and V2 regions increases viral sensitivity to neutralizing antibodies. The V3 loop is exposed and engages the coreceptor (CCR5 or CXCR4), which then mediates membrane fusion. Further studies are needed to better understand how the 136 and 316 sites (HBX2) affect viral transmission.

Broad and potent HIV-1 neutralizing antibodies (bNAbs) are the goal of many HIV-1 vaccine programs [21]. The four most vulnerable sites on the *env* glycoprotein are the CD4 binding site (CD4bs), glycan dependent epitopes in V1V2 and near the base of V3/C3, and linear epitopes in the membrane proximal external region (MPER) of gp41 [21]. These sites are widely recognized to differ between HIV-1 subtypes. Here, we demonstrated that they can also differ between transmissions, which add to the difficulty of developing an effective vaccine.

In summary, we conducted a large-scale sequence analysis of the HIV-1 CRF01_AE. The CRF01_AE sequences isolated in MSM in China formed two clusters and the highest rates of evolution were observed in the *env* gene. In addition, the amino acids mutations at PSS differed between the clusters and are likely associated with virus budding and antigen recognition. These results further our knowledge of CRF01_AE evolution across transmissions, and will likely help improve HIV-1 surveillance and vaccine development.

## Supporting Information

S1 Fig Three distinct phylogenetic clusters of HIV-1 CRF01_AE were identified in China. (A) Phylogenetic tree of the *gag* (HXB2: 796–2216 nt). (B) Phylogenetic tree of the *pol* gene (HXB2: 2085–5096 nt).
(TIF)

S1 File The accession numbers for the sequences used in this study. (XLS)

## References

[1] Beyrer C, Baral SD, van Griensven F, Goodreau SM, Chariyalertsak S, Wirtz AL, et al Global epidemiology of HIV infection in men who have sex with men. Lancet. 2012;380(9839):367–77. 10.1016/S0140-6736(12)60821-6 22819660 PMC3805037

[2] Hall HI, Song R, Rhodes P, Prejean J, An Q, Lee LM, et al Estimation of HIV incidence in the United States. Jama. 2008;300(5):520–9. 10.1001/jama.300.5.520 18677024 PMC2919237

[3] Birrell PJ, Gill ON, Delpech VC, Brown AE, Desai S, Chadborn TR, et al HIV incidence in men who have sex with men in England and Wales 2001–10: a nationwide population study. The Lancet Infectious diseases. 2013;13(4):313–8. 10.1016/S1473-3099(12)70341-9 23375420 PMC3610092

[4] China. MoHotPsRo. China 2010 UNGASS Country Progress Report (2008–2009). UNGASS. 2010;http://data.unaids.org/pub/Report/2010/china_2010_country_progressreport_en.pdf.

[5] Han X, An M, Zhang M, Zhao B, Wu H, Liang S, et al Identification of 3 distinct HIV-1 founding strains responsible for expanding epidemic among men who have sex with men in 9 Chinese cities. Journal of acquired immune deficiency syndromes. 2013;64(1):16–24. 10.1097/QAI.0b013e3182932210 23542640 PMC3814940

[6] Zhang X, Li S, Li X, Li X, Xu J, Li D, et al Characterization of HIV-1 subtypes and viral antiretroviral drug resistance in men who have sex with men in Beijing, China. Aids. 2007;21 Suppl 8:S59–65. 10.1097/01.aids.0000304698.47261.b1 . 18172393

[7] Wang W, Xu J, Jiang S, Yang K, Meng Z, Ma Y, et al The dynamic face of HIV-1 subtypes among men who have sex with men in Beijing, China. Current HIV research. 2011;9(2):136–9. . 21361866 10.2174/157016211795569096

[8] Feng Y, He X, Hsi JH, Li F, Li X, Wang Q, et al The rapidly expanding CRF01_AE epidemic in China is driven by multiple lineages of HIV-1 viruses introduced in the 1990s. Aids. 2013;27(11):1793–802. 10.1097/QAD.0b013e328360db2d 23807275 PMC3819312

[9] Stamatakis A. RAxML version 8: a tool for phylogenetic analysis and post-analysis of large phylogenies. Bioinformatics. 2014;30(9):1312–3. 10.1093/bioinformatics/btu033 24451623 PMC3998144

[10] Drummond AJ, Suchard MA, Xie D, Rambaut A. Bayesian phylogenetics with BEAUti and the BEAST 1.7. Molecular biology and evolution. 2012;29(8):1969–73. 10.1093/molbev/mss075 22367748 PMC3408070

[11] Minin VN, Bloomquist EW, Suchard MA. Smooth skyride through a rough skyline: Bayesian coalescent-based inference of population dynamics. Molecular biology and evolution. 2008;25(7):1459–71. 10.1093/molbev/msn090 18408232 PMC3302198

[12] Drummond AJ, Ho SY, Phillips MJ, Rambaut A. Relaxed phylogenetics and dating with confidence. PLoS biology. 2006;4(5):e88 10.1371/journal.pbio.0040088 16683862 PMC1395354

[13] Pond SL, Frost SD, Muse SV. HyPhy: hypothesis testing using phylogenies. Bioinformatics. 2005;21(5):676–9. . 15509596 10.1093/bioinformatics/bti079

[14] Yang Z. PAML 4: phylogenetic analysis by maximum likelihood. Molecular biology and evolution. 2007;24(8):1586–91. 10.1093/molbev/msm088 . 17483113

[15] Yang Z, Bielawski JP. Statistical methods for detecting molecular adaptation. Trends in ecology & evolution. 2000;15(12):496–503. . 11114436 10.1016/S0169-5347(00)01994-7 PMC7134603

[16] Sergei L KPA, Simon DW. Frost: Estimating selection pressures on alignments of coding sequences Analyses using HyPhy 2007. [http://wwwhyphyorg/pubs/hyphybook2007pdf]. 2007.

[17] Yang Z, Wong WS, Nielsen R. Bayes empirical bayes inference of amino acid sites under positive selection. Molecular biology and evolution. 2005;22(4):1107–18. 10.1093/molbev/msi097 . 15689528

[18] Rangel HR, Weber J, Chakraborty B, Gutierrez A, Marotta ML, Mirza M, et al Role of the human immunodeficiency virus type 1 envelope gene in viral fitness. Journal of virology. 2003;77(16):9069–73. 12885922 10.1128/JVI.77.16.9069-9073.2003 PMC167250

[19] Pancera M, Zhou T, Druz A, Georgiev IS, Soto C, Gorman J, et al Structure and immune recognition of trimeric pre-fusion HIV-1 Env. Nature. 2014;514(7523):455–61. 10.1038/nature13808 25296255 PMC4348022

[20] Cicala C, Arthos J, Fauci AS. HIV-1 envelope, integrins and co-receptor use in mucosal transmission of HIV. Journal of translational medicine. 2011;9 Suppl 1:S2 10.1186/1479-5876-9-S1-S2 21284901 PMC3105502

[21] Klein F, Mouquet H, Dosenovic P, Scheid JF, Scharf L, Nussenzweig MC. Antibodies in HIV-1 vaccine development and therapy. Science. 2013;341(6151):1199–204. 10.1126/science.1241144 24031012 PMC3970325

