# Supplementary figures and images for: Heterogeneous Evolution of HIV-1 CRF01_AE in Men Who Have Sex with Men (MSM) and Other Populations in China
Source: PLoS One. 2015 Dec 1;10(12):e0143699. doi: 10.1371/journal.pone.0143699 (PMC4666620; doi:10.1371/journal.pone.0143699)

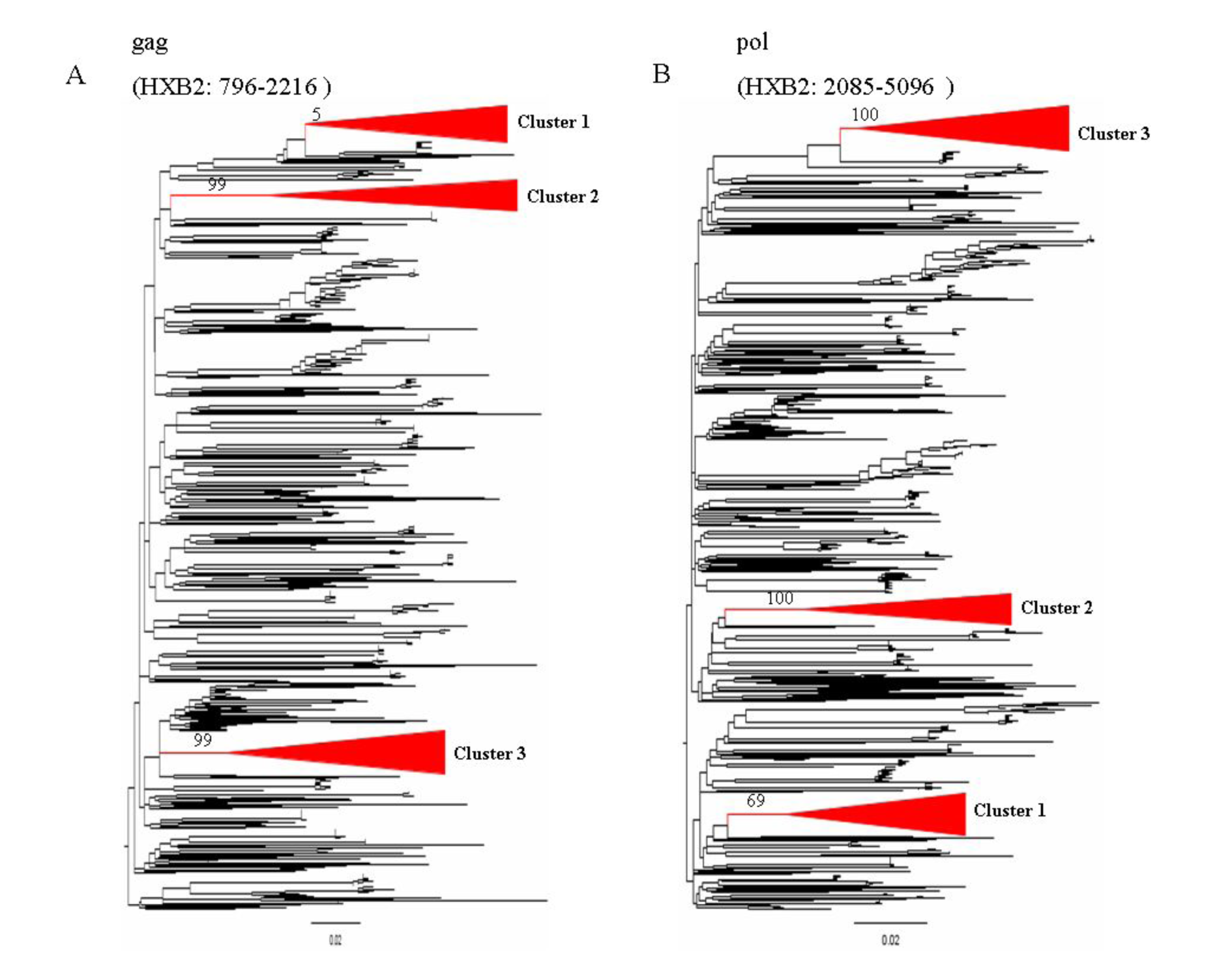

Supplement: S1 Fig — (A) Phylogenetic tree of the gag (HXB2: 796–2216 nt). (B) Phylogenetic tree of the pol gene (HXB2: 2085–5096 nt). (TIF) [file pone.0143699.s001.tif]
